# MiST: A new approach to variant detection in deep sequencing datasets

**DOI:** 10.1093/nar/gkt551

**Published:** 2013-07-04

**Authors:** Sailakshmi Subramanian, Valentina Di Pierro, Hardik Shah, Anitha D. Jayaprakash, Ian Weisberger, Jaehee Shim, Ajish George, Bruce D. Gelb, Ravi Sachidanandam

**Affiliations:** ^1^Department of Genetics and Genomic Sciences, Icahn School of Medicine at Mount Sinai, 1425 Madison Avenue, NY 10029, USA, ^2^The Mindich Child Health and Development Institute, Icahn School of Medicine at Mount Sinai, 1425 Madison Avenue, NY 10029, USA and ^3^Department of Pediatrics, Icahn School of Medicine at Mount Sinai, 1425 Madison Avenue, NY 10029, USA

## Abstract

MiST is a novel approach to variant calling from deep sequencing data, using the inverted mapping approach developed for Geoseq. Reads that can map to a targeted exonic region are identified using exact matches to tiles from the region. The reads are then aligned to the targets to discover variants. MiST carefully handles paralogous reads that map ambiguously to the genome and clonal reads arising from PCR bias, which are the two major sources of errors in variant calling. The reduced computational complexity of mapping selected reads to targeted regions of the genome improves speed, specificity and sensitivity of variant detection. Compared with variant calls from the GATK platform, MiST showed better concordance with SNPs from dbSNP and genotypes determined by an exonic-SNP array. Variant calls made only by MiST confirm at a high rate (>90%) by Sanger sequencing. Thus, MiST is a valuable alternative tool to analyse variants in deep sequencing data.

## INTRODUCTION

Whole-exome sequencing (WES), mRNA-seq and whole-genome sequencing are amongst several commonly used techniques based on deep-sequencing that allow extensive sampling of the genome to detect variants. WES samples the exonic parts of the genome through physical capture of the relevant sequences (1). This is a lower-complexity alternative to whole-genome sequencing, as only 3–4% of the genome is sampled and directs attention only to the coding regions.

There are several software pipelines that analyse the data from WES including GATK (2,3), Samtools (4), Freebayes (5) and Bambino (6). Broadly, their approach involves mapping the sequences to the reference genome to generate BAM/SAM files. These alignment files are subsequently analysed to infer variants and SNPs (7). In contrast to the extant tools, MiST closely mimics the experimental technique, using exonic sequences as bait to identify sequences that can potentially map to the exon. A subsequent fine-mapping step, which aligns the selected reads against the exonic regions, permits a more sensitive and accurate identification of SNPs and variants. This approach reduces the computational complexity and allows for more sensitive mapping.

For WES data from one sample, we compared MiST variant calls to those from GATK (2,3) and a genotyping micro-array. We picked GATK due to its popularity. The MiST calls exhibit greater concordance with the genotyping array results and entries in dbSNP. The algorithm and pipeline are described in the Material and Methods section. We provide a detailed comparison of the programs and highlight similarities and differences for selected variants. We conclude by highlighting the salient features in the discussion and justify the development of an alternative variant-calling platform.

## MATERIALS AND METHODS

### Samples

We obtained genomic DNA from an anonymous subject, M01_1. The sample was prepared and sequenced by standard techniques, described below.

### Sample prep and sequencing

The samples were prepared using a capture kit from Nimblegen (the first version) using standard procedures recommended by the manufacturer. The process was optimized to reduce clonality, by controlling the number of PCR cycles used for amplification in various steps. The sequencing data consists of paired-end reads from an Illumina HiSeq run (usually 2 × 100 nt, 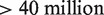 reads per sample). We used the hg19 version of the human genome for the annotations, which consists of 120 000 exons (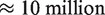 nucleotides).

### Exonic SNP arrays

The Infinium HumanExome BeadChip from Illumina, which has 240 000 exonic markers and 700 000 genome-wide markers, was used to genotype the sample.

### Computational infrastructure

The hardware is built from off-the-shelf components. We currently use a cluster of 10 nodes, each with two processors, four cores per node. Each node has 32 GB of RAM. The nodes share a 10-TB storage, which holds data and the results of the analysis. The nodes are connected to the storage through a 10 GB/sec switch. We have also run the software on a single computer with 128-GB RAM, 6 TB of disk space and four CPUs (AMD Opteron Processor 6220) with eight cores each.

### Software platforms

We use MySQL databases and Apache webservers installed on the Ubuntu Linux operating system. Our software consists of mostly custom-built mix of back-end code written in Perl, C, C++ and Python, and front-end user-interfaces written in Perl, PHP, Python and JavaScript.

### The pipeline

**Figure 1.**
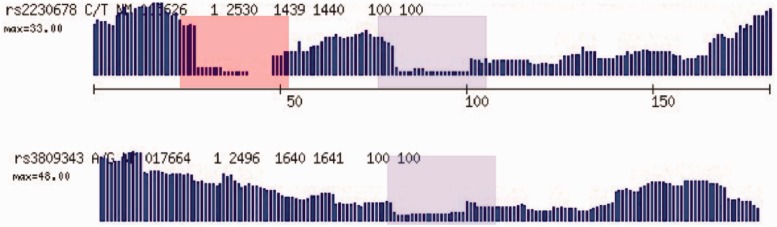
Motivation for the use of Geoseq in variant calling. When exact matches of tiles from mRNAs in the sequenced reads are plotted, SNPs in the query sequence lead to gaps of the size of tiles in the matches. We see here examples of homozygous (top panel) and heterozygous (bottom panel) SNPs. This suggests that alignment of reads that map to the gaps can identify SNPs and indels.

**Figure 2.**
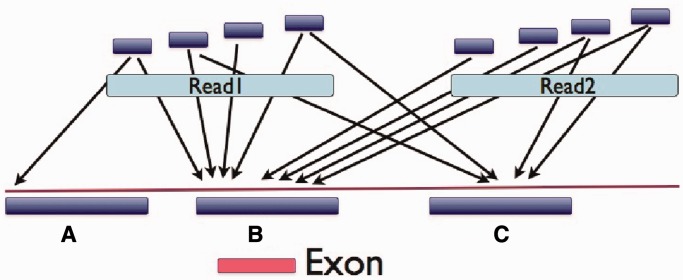
Identification of potentially paralogous read pairs. Perfect matches are found in the genome for tiles from each read in a pair. A majority rule identifies the origin of the read pair. In this case region B is identified as the origin of the read-pair which includes the target exon shown below it. So, the read pair is selected for careful alignment to the target exonic region. This process can distinguish between pseudo-genes and their partner genes, as well as homologous genes since, for most exons, at least one member of the pair will extend into introns which evolve at a neutral rate and exhibit differences specific to their genomic location.

We motivate our method by using Geoseq to analyse mRNA-seq data. Geoseq identifies reads with exact matches to tiles from the query sequence. The graph in Figure 1 shows matches to 25-nt tiles from the mRNA in the reads. Variations from the reference sequence cast *shadows* on the graphs as holes in the coverage. This suggests that a fine mapping of reads that map to the shadow can accurately estimate the allele frequencies in the samples. The reads are searched for exact matches to tiles from a chosen exonic region using Geoseq (8). Smaller tiles will allow more mismatches, making it more sensitive but increasing the computational cost for aligning the reads. Empirically, we determined 25 nt to be an optimum tile size (Table 1). Reads that map ambiguously or to multiple locations on the genome are then eliminated by the process depicted in Figure 2, and the surviving reads are aligned to the exonic region using BLAST (9). The alignments are analysed to remove clonal reads and to infer variants and indels.

**Table 1.** The effect of tile-size on Geoseq results

Tile size	Sequences retrieved	System time (in seconds)
15	7049	0.52
18	1135	0.54
20	74	0.51
21	32	0.52
25	14	0.52
28	14	0.48
30	4	0.5

The tile size determines the number of reads selected for further analysis, which determines sensitivity, specificity and speed. Reads were retrieved using Geoseq with different tile sizes for a 1158-nt-long terminal exon from a variant of *ASB10* (chr7:150883831-150884989). There is a sharp transition in the number of reads retrieved at a tile-size of 20; at lower tile sizes a majority of the retrieved reads map to multiple locations on the genome. We picked 25 as an optimum tile size based on an expected average of 1 variant per 100 nt in the genome. The time to align the retrieved reads against the exons grows linearly in the number of sequences.

We split the sequenced reads into randomly assorted groups (four or more) to parallelize the processing and ensure the index files can fit on RAM. A file system on ramdisk is used to store the data being processed, reducing the file input/output (I/O) overhead, which can affect performance. A relational database (MySQL) is used to store tabular data, for rapid access to information, which is used by browser-based tools for data filtering. The variants are inferred from the alignments and annotated for the effects on the protein (missense, nonsense, stop codon etc.) using ANNOVAR (10). The variants are marked with dbSNP and 1000 Genomes annotations. SIFT (11) and SNAP (12) are used to determine coding variants with deleterious effects.

**Figure 3.**
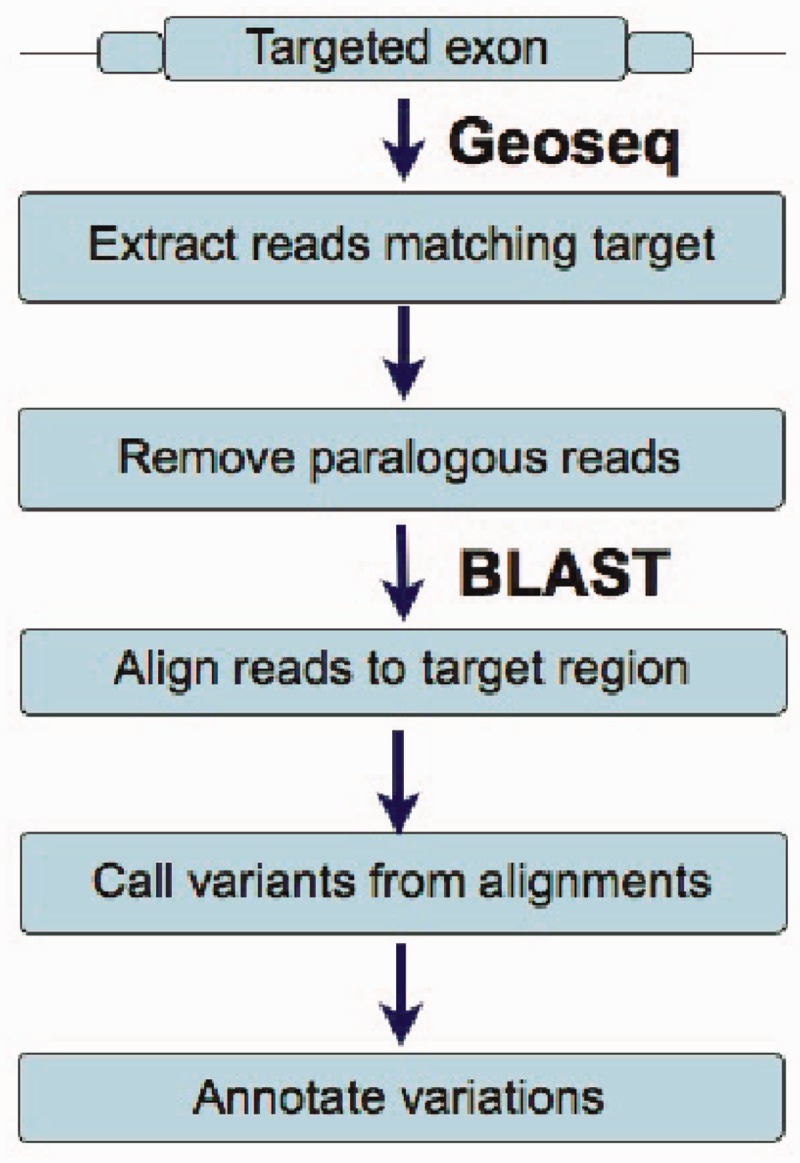
A schematic of the workflow used by MiST. The first step retrieves reads that can potentially match a targeted exonic region, and the following steps remove reads that can match other locations on the genome and align the reads to the exonic regions to call variants from the reference sequence.

The various steps in our pipeline depicted in Figure 3 can be grouped into the sub-categories listed below.

### Reference sequence pre-processing

A reference set of exonic sequences is collected by first starting with the sequences of exons of RefSeq mRNAs from the human genome (hg19) and other exons from the targets of the capture kit. Each exon is extended by 70 bp on the 5′ and 3′ ends to capture reads that can reach into introns. The extension is a function of the average insert size, which is determined by the experimental protocol. Overlapping exons are merged into super-exons. Each exon is indexed for BLAST searches. The human genome is indexed for BLAST as well as suffix-array searches by Geoseq.

### Fastq pre-processing

Reads containing ambiguous bases (usually marked as Ns) or with stretches of poor sequence quality (any 16-nt window with an average quality score <10) are removed. If either of the reads in a read pair fails the quality check, the pair is discarded. Each surviving read pair is then concatenated into a single string. This set of concatenated read pairs is split into four (or more) subsets and each subset is indexed using the Geoseq suffix array indexer. The splitting allows parallelizing the process and reduces the size of the indices so they can fit into RAM on a single processing node. We do not consider the quality scores beyond this step.

### Sequence retrieval and map filtering

For each of the reference exonic region, matching reads are retrieved using Geoseq with 25-nt tiles (Table 1).

Matching reads are validated by identifying the approximate locus for each read pair and keeping only those read pairs that originate within a window (determined by the insert size) of the ends of the target exon. The approximate location is determined as follows (Figure 2):

- Up to four non-overlapping tiles (subsequences) with the same length as the Geoseq word size are chosen from each read in a pair. Tiles are discarded if they contain stretches of mononucleotide or dinucleotide repeats. The suffix-array index of the reference genome is used to retrieve all mapped positions for each tile.
- Tiles with greater than five potential mappings to the genome are discarded. If no tiles remain, then the read pair is discarded. For the remaining tiles, a 500-nt window around each of the mapping positions is marked as the potential origin of the read pair.
- A match number is assigned to each window. The match number is the number of tiles that map to the window. The window with the maximum match number is deemed to be the origin of read pair. If multiple windows share the same maximum match number, then the read pair is discarded.
- If the selected window does not contain an end of the targeted exon, the read pair is discarded.

The surviving read pairs are split into their constituent reads and placed consecutively in a fasta file.

### Alignment of reads to genomic fragments

**Figure 4.**
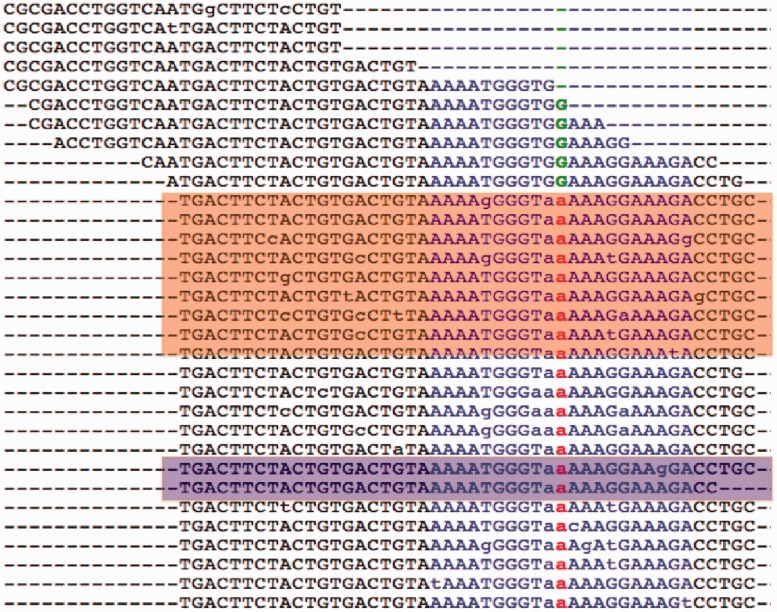
Clonal reads arise from multiple sequencing of the same clone due to biased PCR amplification of the samples. Clonal reads are identified more reliably with paired-end sequencing. The orange box shows a set of clonal reads that map to the same stretch of the genome with a few mismatches within the reads. Even with poor sequencing quality, by requiring that at least three out of four ends of the pairs coincide, clonal reads are reliably identified. The violet box shows clonal reads with one varying end due to poor sequence quality. The corrected coverage of the variant shown in the figure (in red) is 8 (A-2/G-6), while the original coverage is 241 (A-201/G-40). Clonality causes a spurious increase in coverage, creating erroneous variant calls, and an overestimation of the quality of capture and sequencing.

**Figure 5.**
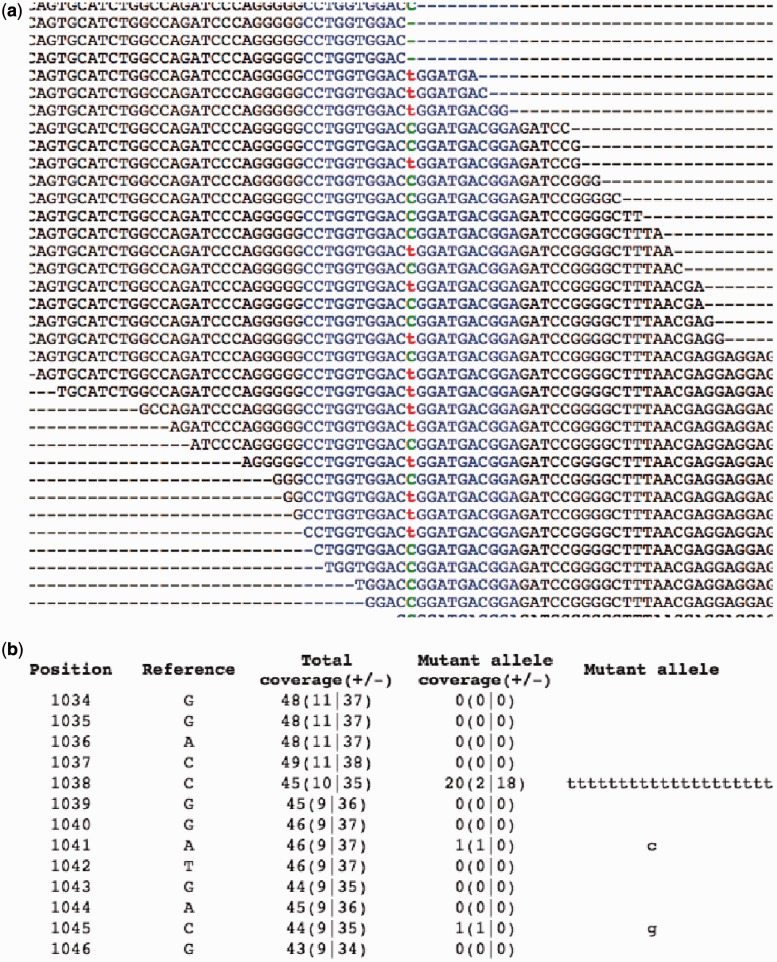
Only MiST detected the variant in gene NLRC3, R282W (chr16:3614094 on hg19), which was confirmed by Sanger sequencing. (**a**) Alignment View and (**b**) Pileup View. In the pileup view, column 1 is the position relative to the start of the genomic fragment, column 2 is the reference allele, column 3 gives coverage at that position, with the number of reads in forward and reverse directions (±) shown within parenthesis, column 4 gives the coverage for the mutated allele and the non-reference allele in the reads are shown in column 5. The name of the file contains the position of the fragment in the genome. MiST calls this SNP despite a strong skew (strand-bias) in the mutant allele because the reference allele also shows a strong skew.

The fasta file of selected reads is aligned against the exonic sequence, using the legacy engine (the option -V T) of BLAST, to create an accurate alignment (Figure 4). The alignments (Figure 5a) are processed to build a pile-up file (Figure 5b) that annotates each base in an exon with features such as number of reads aligned on the positive and negative strands and mismatches with strand information.

### Variant calling

We used a low threshold on coverage and mutation frequency for calling variants in a trio (parents and child) to identify candidate variants. The use of a trio allows for more robust identification of false positives in the *de novo* calls by identifying potential parental contributions. Using Sanger sequencing of the data from trios (parents and child), we found that the false positives predominated when the coverage was below 15 and/or the minor allele frequency dropped below 0.3, which is our threshold for variant calling. For each variant, as the sequencing is independent of the strand, the contributions from the two strands are expected to be equal. The imbalance in the contributions from the two strands is measured by strand skew, defined as 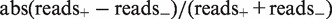 where 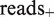 is the number of reads on the plus strand. A strong difference in strand skew between the reference and variant alleles is a good indicator of erroneous calls (Figure 5b). Clonal reads are identified and removed at this stage for calculating variant statistics, but are shown in the alignments (Figure 4). The reference exon id, coverage, mutation frequency, mutant and reference alleles, genomic position, and immediate 21-nt flank (10 nt on either side of the variant) are recorded for each predicted variant. Mappings of the 21-nt flank on the reference genome are annotated to identify potential paralogous variants.

### Variant annotation

The position of the variant within each overlapping RefSeq mRNA is recorded along with the specific mRNA region (intron, UTR, CDS, etc). For CDS variants, the effect of the mutation on the amino-acid sequence is predicted using the known open reading frame for each Refseq mRNA. We identify variants in our list that occur in dbSNP, 1000 Genomes or private collections to highlight the novel variants that are of interest in most studies of rare genetic disorders. The variants are identified by their flanking sequence, 10 nt on each side. Variants and their host genes with known disease/phenotype associations in dbGAP (13), OMIM (omim.org), PhenCode (14), SNPedia (15) and PharmGKB (16) are annotated. Missense variations are checked against the Polyphen predictions made on the UniProt protein database for severity of the mutation. Variants are also checked for their effect on protein function using a local installation of SNAP (12). These annotations, used for filtering variants, are placed in a database. The pipeline can also generate results in commonly accepted formats for sequence variants and alignments like Variant Call Format (VCF) and SAM/BAM file formats.

The variants can be filtered based on coverage, gene name, skew, effect of variant (missense, nonsense), prevalence in child versus parents, pathways, diseases and SNP associations to disorders. The raw data, such as coverage over exons in a gene, alignments (Figure 4), pileup reports (a synopsis of the full alignments, Figure 5) can also be explored to develop a better understanding of the variants and the underlying evidence.

## RESULTS

We processed exome sequencing data from a sample with GATK as well as MiST. The sample was also genotyped on a Human Exome SNP array. The GATK pipeline was the one implemented at the Yale Genome Center. We compare and contrast our results with GATK as a means to place them in context.

There are differences in variant calls made by the two pipelines. We list them in different categories and give an explanation for each one. A few caveats must be emphasized in any such comparison. The first one is that the performance of programs are best tuned by experts in the program, thus comparisons can be faulted for not having the optimal conditions. Another caveat is that all tools, by their very nature, are moving targets and undergo constant evolution with improvements being added in response to reviews and reports from the field. We are offering a snapshot view in this comparison.

**Figure 6.**
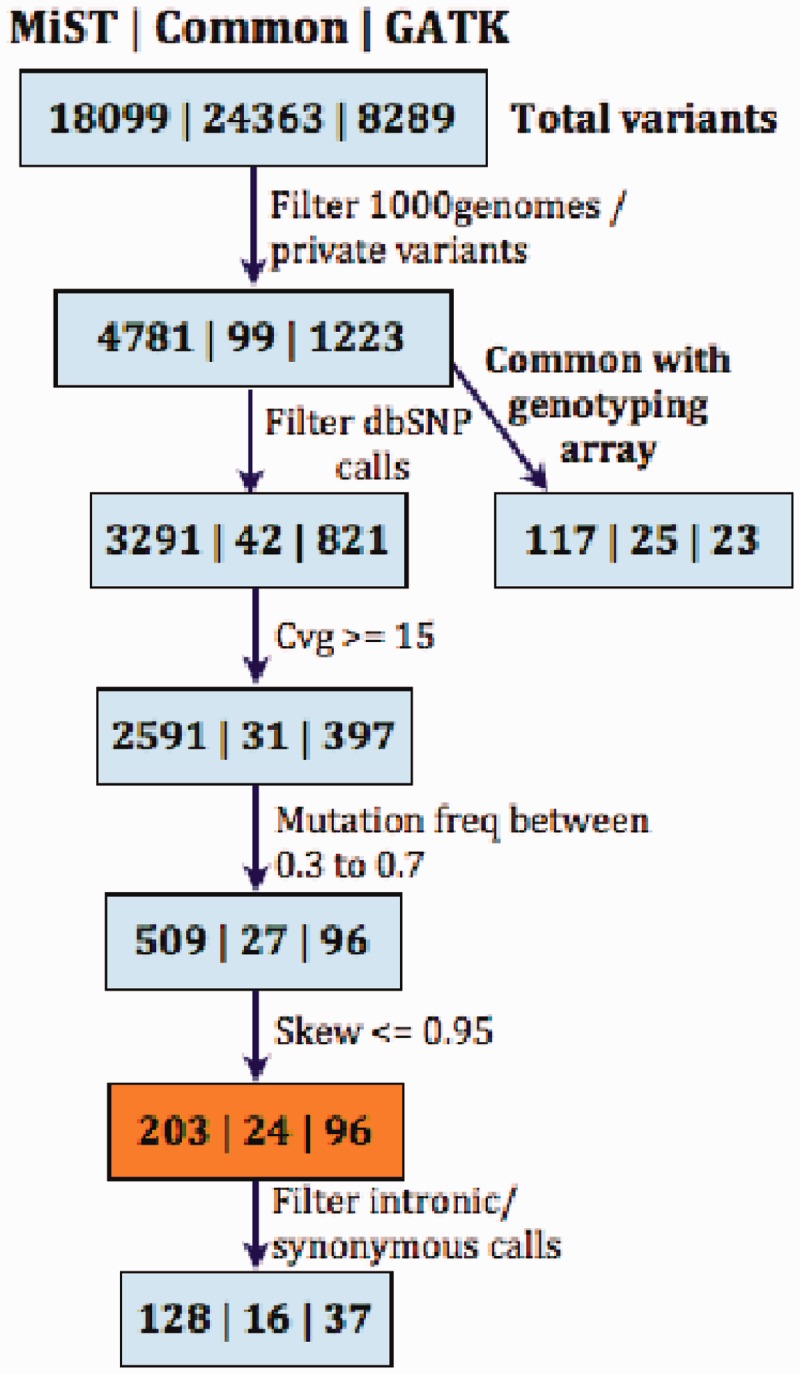
Comparison of MiST and GATK. Each box has three sets of numbers, from left to right they are variant calls, (i) unique to MiST, (ii) common to both platforms and (iii) unique to GATK. Filters are applied to remove calls occurring in public databases like dbSNP (17), 1000 Genomes (18) and a collection of already known private variants. MiST called 14 808 variants from dbSNP and 1000 genomes as opposed to 7468 variants by GATK. MiST had more variants in common with the exonic genotyping array, compared with GATK. In the box shaded orange, of the 96 calls unique to GATK, 25 calls map to multiple locations, 35 calls were far from exonic boundaries, 6 calls were eliminated by MiST for arising in low complexity regions such as a run of T’s, 14 calls were eliminated by MiST due to clonality corrections. In addition, there were 16 calls private to GATK (7 in UTRs, and 9 synonymous calls) that were not called by MiST, because the exons are not present in RefSeq.

**Figure 7.**
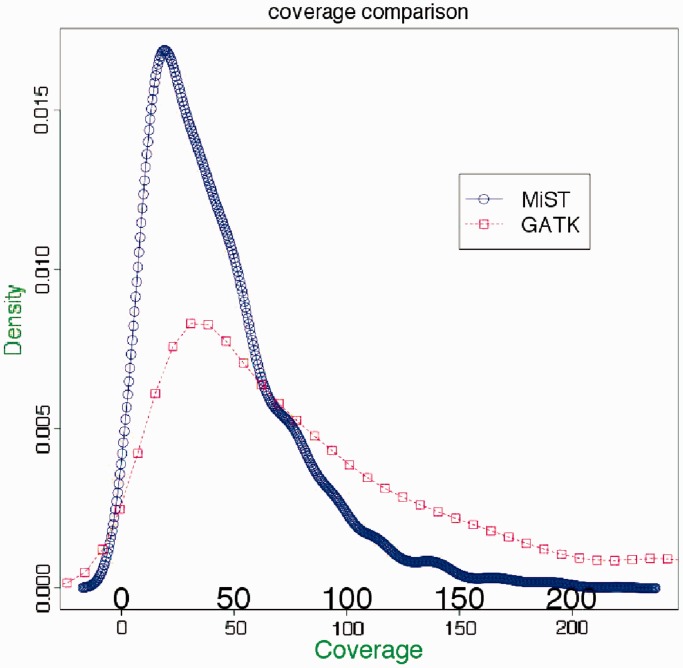
A comparison of coverage between the MiST and GATK pipelines. The graph shows density distributions of coverage over variants that have been called by both platforms. The total area under each curve is 1. As seen from the graph, MiST has, on average, lower coverage per variant compared with GATK, due to more stringent removal of artifacts arising from clonal reads as well as reads that map to multiple locations.

We started out with >42 000 variant calls, containing some poor-quality calls that were left in to assess the effect of various filters (Figure 6). MiST stringently filters paralogous reads, avoiding those mapping to multiple genomic locations. There are a number of dbSNP entries that occur in these paralogous regions, which we believe are erroneous calls. An example is 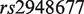 with coverage of 765 and a minor allele frequency of 0.31. The 21-nt flank of this SNP (10 nt on either side) maps to 35 locations on the genome. MiST excludes this, while GATK calls this SNP. Our pipeline also corrects for clonal reads, by removing them from consideration for the calculation of coverage and calling variants (Figure 4). Owing to these corrections, MiST calls often have lower and more accurate coverage compared to GATK (Figure 7).

We compare and contrast several features in GATK and MiST:

- Total number of variants. MiST calls more variants than the GATK pipeline, but this is a function of the range of coverage and minor-allele frequencies that are permitted. MiST has more in common with dbSNP and the SNP genotyping array. Figure 6 shows the effects of applying various filters to the calls from MiST and GATK. We now explain the variants unique to each of the programs.
  - Novel variant calls in GATK, but not MiST. The final list from GATK had 96 heterozygous missense calls not called by MiST. Most of these were eliminated by the paralogous read filter in MiST. For example, in the gene *GGT1* (chr22 at 25023513), the flanks mapped exactly to 5 locations on the genome. In another variant, in *JMJD8* (chr16 at position 733735), the reads mapped to multiple locations. In both cases, the coverage fell below threshold after removing reads with multiple mappings.
  - Novel variant calls by MiST but not GATK. We initially pursued two such variants, (1) in the gene *NLRC3* (at chr16-3614094,C >T, aa R282W) with a coverage of 45 and allele frequency of 0.444. This was also detected on the array with a B-allele frequency of 0.533 (Figure 5) and (2) in the gene *PTCHD3* (at chr10-27687638, G >A, R630Q, coverage of 33 and minor allele frequency of 0.363). This was also detected on the array with a B-allele frequency of 0.505. Both were confirmed by Sanger sequencing. They were missed by GATK due to incorrect reference allele coverage which is inflated by the clonal reads.
  To test the quality of MiST calls in an unbiased manner using Sanger sequencing, we picked a panel of 27 high-quality variant calls unique to MiST, consisting of a mix of heterozygous and homozygous SNPs (Table 2 and Supplementary Table S1).
    Twenty-three of them confirmed while two failed at the PCR step and two failed to show the expected alleles on sequencing (Supplementary Figure S1).
- Coverage on calls. We expect lower coverage on calls on average from MiST, compared with GATK, due to the stringent handling of clonal/paralogous reads. This was confirmed by the distribution of coverage across variants common to both platforms (Figure 7).
- Transitions (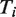) versus transversions (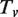). The ratio 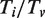 is expected to be 2.0 for neutral SNPs. In coding regions, this ratio has been empirically shown to be closer to 3.0 (19). The majority of the variant calls are common to the two programs, which does not allow for major differences in these measures. For SNPs with high coverage (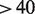) and frequencies in the range of 0.35–0.65, the non-synonymous SNPs exhibit a ratio of 2.11 (GATK 2.22) and the synonymous SNPs exhibit a ratio of 5.04 (GATK 6.06).

### Performance comparison

We used MiST and GATK on the same sample using the same computer to compare the performance of the two pipelines. The analysis was performed on a Linux computer (Ubuntu) with four 8-core CPUs (32 cores total) and 128 GB of RAM. A paired-end sample with 47 million reads was analysed using both pipelines on 2721 genes with a total of 25 034 exons. Variant calling using MiST took a total of 5 h while GATK took 9 h.

**Table 2.** A mix of heterozygous and homozygous SNPs called by MiST but missed by GATK were selected for confirmation by Sanger Sequencing

Class	Total	PCR fail	Sanger fail	Sanger Success
MiST	19	2	1	16
MiST + Array	8	0	1	7

They also exhibited a range of effects (missense, nonsense, non-coding etc.), and some were also part of the panel targeted by the genotyping array. The detailed breakdown is given in Supplementary Table S1.

The breakdown for various processes in MiST are as follows: indexing reads for Geoseq (22 min), identifying reads targeting exonic regions (135 min), removal of paralogous reads (74 min), read alignments to exonic regions and variant calling (63 min).

Using GATK with the best practice variant detection method suggested on their website, the time taken for different phases of variant detection method is as follows. Phase I, the raw data processing, took 130 min to generate alignments in a SAM file using BWA, 62 min for sorting SAM and creating BAM using Picard, 33 min for adding read groups to sorted BAM using Picard, 45 min to mark PCR duplicates, 116 min to generate local realignments around indels, 52 min for realignment and 58 min for using Fixmate for paired-ends. Phase II took 56 min to produce the RAW SNP calls.

## DISCUSSION

MiST is a radically different approach to variant calling, compared with other tools and approaches that are in use. Having different approaches is useful, as a diversity of techniques will lead to more robust variant calls through the use of a *wisdom of the crowds* approach (20). Even in experiments, different sequencing platforms (Complete Genomics, Illumina HiSeq) and capture techniques (Nimblegen, Agilent) (21) exhibit different variant calls and biases.

The process outlined in the article is easy to implement and provides a different viewpoint to SNP calling. The software allows users to explore the data generated at every step, as they are available in plain text formats. MiST is more sensitive and computationally efficient. This effective technique is well-suited for other datasets such as targeted resequencing and mRNA-seq.

MiST does not use quality scores, except to filter out reads that have stretches of poor quality nucleotides, as they are inaccurate, difficult to correct and change with instrument upgrades. At the coverage required for variant calling (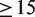), it is highly unlikely that a genomic location will show the same error more than once in non-clonal reads (the probability of Q30 bases from two reads having a coincident error is <1 in 10 000, and even smaller if multiple reads are considered). This improves computational efficiency.

MiST carefully weeds out paralogous/repeat mapping, reducing a major source of error in variant calls. It also carefully corrects for clonal reads, not just considering exact matches, but also using mapping to identify clones in poor-quality reads as shown in Figure 4. The accuracy of MiST calls is attested to by the comparisons with GATK. MiST does not require specialized hardware and is suitable for small-scale applications.

MiST is well-suited for studies involving limited areas of the genome, even if the data are from whole-genome sequencing. MiST does not provide any advantage in cases such as cancer-genome sequencing, where mapping to the whole genome is essential. In genomic regions with high variability, such as areas of kataegis (22), traditional local alignment methods using algorithms such as Smith–Waterman will be more suitable. To determine the suitability of MiST for their needs, researchers will need to compare its results with those from several tools on a representative set of data, as tools are constantly evolving and tend to differ in their areas of excellence.

## CONCLUSION

MiST is an alternate approach that is efficient and sensitive for variant detection in deep sequencing datasets. MiST works well on both single and paired-end data from whole-exome capture and sequencing. MiST is easy to implement and can be tailored to changes, such as insert sizes, in the experimental protocol. MiST adds a novel method to the variant calling marketplace, allowing investigators to compare and contrast the results with other more commonly used platforms.

## SUPPLEMENTARY DATA

Supplementary Data are available at NAR Online: Supplementary Table 1 and Supplementary Figure 1.

## FUNDING

Seed fund from The Icahn School of Medicine at Mount Sinai (to R.S.). Partial funding from National Institutes of Health [U01 HL098123 to R.S. and B.D.G.]. Funding for open access charge: Pilot fund from the Children’s Environmental Health Center at The Icahn School of Medicine.

*Conflict of interest statement.* None declared.

## Supplementary Material

Supplementary Data
