# Supplementary material for: MiST: A new approach to variant detection in deep sequencing datasets
Source: Nucleic Acids Res. 2013 Jul 4;41(16):e154. doi: 10.1093/nar/gkt551 (PMC3763541; doi:10.1093/nar/gkt551)
Supplement: Supplementary Data [file supp_41_16_e154__index.html]

MiST: A new approach to variant detection in deep sequencing datasets — MiST: A new approach to variant detection in deep sequencing datasets — Supplementary Data 

# MiST: A new approach to variant detection in deep sequencing datasets

## 

files

**Files in this Data Supplement:**

- Supplementary Data - pdf file
