# Supplementary material for: MiST: A new approach to variant detection in deep sequencing datasets
Source: Nucleic Acids Res. 2013 Jul 4;41(16):e154. doi: 10.1093/nar/gkt551 (PMC3763541; doi:10.1093/nar/gkt551)
Supplement: Supplementary Data [file supp_gkt551_nar-01148-met-n-2013-File013.pdf]

SUPPLEMENTARY MATERIAL

Supplementary material for *MiST: A new approach to variant detection in deep sequencing datasets* .

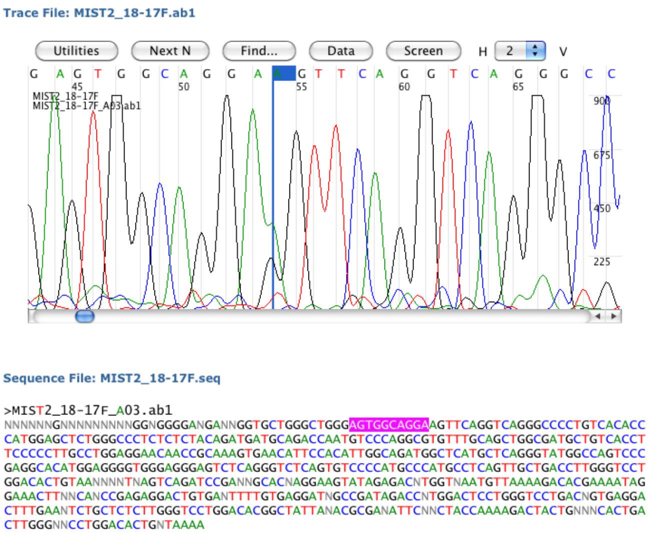

(a) AIFM3

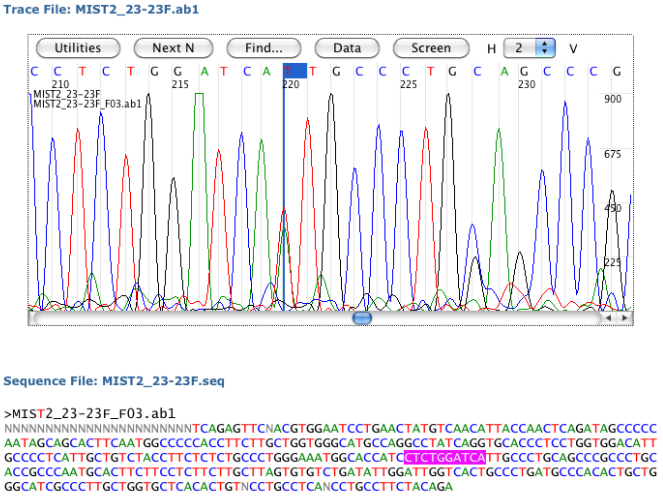

(b) OR51S1

. **Supplementary Figure S1**. Sanger results for two MiST calls (out of the 27 listed in Supplementary table 1) that were not identified by the GATK pipeline and confirmed by Sanger sequencing.

. **Supplementary Table S1**. SNPs private to MiST, checked by Sanger sequencing. Table 1 is a synopsis of this data. The MSSM column indicates if the variant has been seen in other samples processed by MiST. The frequency column is the allele frequency.

flank	chr	pos	gene	RefSeq	effect	cvg	frequency	skew	sanger	dbSNP132	Array-GT	MSSM
GACAAACCCC[C|-]CATTCCCTTC	16	28993654	SPNS1	NM_001142448	In	62	0.5	0.03	100%-;0C	Novel	-	Known
GACCTACCCT[T|C]TCTGCTGCTT	20	44053069	PIGT	NM_015937	In	96	1	0.08	100%C	rs2741566	-	Novel
ACATCATCCT[A|G]ACAAATGGTC	3	151012497	GPR87	NM_023915	syn	83	1	0.06	100%G	rs2242353	-	Novel
AGGCCTTTGC[T|C]TTCTGCTCCA	1	24201262	CNR2	NM_001841	syn	77	1	0.01	100%C	rs2502993	-	Novel
GGGACGGAGA[T|G]CAAATAAAGG	12	6428116	PLEKHG6	NM_018173	In	69	0.985	0.07	100%G	rs4764497	-	Novel
CCACTCCCCT[G|A]TCCCATGGTG	5	176939688	DDX41	NM_016222	In	126	0.984	0.06	100%A	rs28544348	-	Novel
CTGCTGGCCA[G|Gc]CTGTCGGAGC	16	138774	NPRL3	NM_001039476	ins-fs	108	0.471	0.06	100%C;0-	Novel	-	Known
GTCAAAAGCC[A|G]TAAGCCAGAG	2	113513825	CKAP2L	NM_152515	ms	65	1	0.01	100%G	rs6731822	[C/C]	Novel
GCCTCTCACG[T|C]CTGGGTAATG	9	139699964	KIAA1984	NR_024580	utr	72	0.486	-2	T=385;C=411	rs2811793	-	Novel
GGTGAGAAAA[T|C]GCCAGTCACA	20	21142813	PLK1S1	NM_001163023	ms	95	0.526	0.07	100%T	rs2236178	[T/C]	Novel
CTGGGCTCCC[C|T]GCATCCCCAG	12	56030938	OR10P1	NM_206899	ms	84	0.975	0.05	100%T	rs10876838	[T/T]	Novel
CTTCAGGGTA[C|T]GCCCAGAGCT	12	55523685	OR9K2	NM_001005243	ms	68	0.5	0.09	T=367;C=341	rs12303066	[T/C]	Novel
TAGCAATTCT[G|A]CAAGTTTTAT	1	216595306	USH2A	NM_007123	ms P-P	60	1	0.03	100%A	rs10779261	[T/T]	Novel
AGGAAAAGCA[G|A]GGCTCAGGGC	7	143079806	ZYX	NM_003461	In	69	0.985	0.07	100%A	rs6464546	-	Novel
GGCAGGGAGG[G|A]CTGGAGAGCC	22	26894985	TFIP11	NM_012143	In	99	0.979	0.07	100%A	rs2187956	-	Novel
ATCACGGCTG[T|C]TCCCTGTCCC	7	100695058	MUC17	NM_001040105	In	70	1	0.03	100%C	rs6966570	-	Novel
AGTGGCAGGA[G|A]GTTCAGGTCA	22	21332122	AIFM3	NM_144704	In	122	0.483	0.05	G=218;A=364	rs178277	-	Novel
CCCTCCCTTG[A|G]TAAGTCTCAT	9	35060955	VCP	NM_007126	In	96	1	0.02	100%G	rs2258240	-	Novel
ACTGATGGCC[A|G]CCCCTCTCCT	19	11333596	DOCK6	NM_020812	In	78	0.486	0.03	A=692;G=243	rs3810308	-	Novel
GGCATATGAT[T|C]GTTTCATTGC	11	5345170	OR51B2	NM_033180	ms	66	1	0.03	100%C	rs7952293	[G/G]	Novel
CGGGAGGCCC[A|G]TGCCAAGGCC	11	6221169	OR52W1	NM_001005178	ms	90	1	0.04	100%G	rs10839531	[G/G]	Novel
CTCTGGATCA[T|A]TGCCCTGCAG	11	4870269	OR51S1	NM_001004758	ms	98	0.479	0.04	A=328;T=415	rs12417164	[T/A]	Novel
AGCTTTGTTG[C|A]CGCCTGCAGA	7	100486035	SRRT	NM_015908	In	69	0.478	0.01	A=219;C=124	rs2720392	-	Novel
AGCCTAGCTG[G|A]GCCTTGTCTT	15	41819322	RPAP1	NM_015540	In	81	0.481	0.01	G=535;A=641	rs2297380	-	Novel
GTAAGCCTCT[G|T]CCCCTGTGAT	2	98354139	ZAP70	NM_207519	In	95	0.494	0.05	T=655;G=714	rs17488834	-	Novel
CCCACATCCT[C|T]GCCAGCCACT	15	42132896	PLA2G4B	NM_005090	In	123	0.487	0.04	T=486;C=540	rs1122884	-	Novel
AAGTTCCCAC[G|A]TGTACAGGCC	6	170627651	FAM120B	NM_032448	syn	119	0.478	0.03	PCR-FAIL	rs9460113	-	Novel
TAGAAGTCAG[A|G]TTGGCAGGTG	19	35761519	USF2	NM_003367	In	118	0.491	0.07	PCR-FAIL	rs28365133	-	Novel

Nomenclature:*In* is intronic, *ins* is insertion, *fs* is frameshift , *ms* is missense, *utr* is untranslated, *syn* is synonymous and *P-P* is probably-pathogenic.
